# Blind Identification of Convolutional Encoder Parameters

**DOI:** 10.1155/2014/798612

**Published:** 2014-05-21

**Authors:** Shaojing Su, Jing Zhou, Zhiping Huang, Chunwu Liu, Yimeng Zhang

**Affiliations:** School of Mechatronics Engineering and Automation, National University of Defense Technology, Deya Road, Changsha, Hunan 410073, China

## Abstract

This paper gives a solution to the blind parameter identification of a convolutional encoder. The problem can be addressed in the context of the noncooperative communications or adaptive coding and modulations (ACM) for cognitive radio networks. We consider an intelligent communication receiver which can blindly recognize the coding parameters of the received data stream. The only knowledge is that the stream is encoded using binary convolutional codes, while the coding parameters are unknown. Some previous literatures have significant contributions for the recognition of convolutional encoder parameters in hard-decision situations. However, soft-decision systems are applied more and more as the improvement of signal processing techniques. In this paper we propose a method to utilize the soft information to improve the recognition performances in soft-decision communication systems. Besides, we propose a new recognition method based on correlation attack to meet low signal-to-noise ratio situations. Finally we give the simulation results to show the efficiency of the proposed methods.

## 1. Introduction

In digital communication systems, error-correction codes are widely used. To meet high quality of services, new coding schemes are being developed ceaselessly. Therefore, for a communication receiver, it is very difficult to remain compatible with all standards used. But if it is an intelligent receiver, which is able to blindly recognize the coding parameters of a specific transmission context, it can adapt itself to the perpetual evolution of digital communications. Furthermore, the blind recognition techniques are also applied in noncooperative communications. In noncooperative communication contexts, a receiver does not know the coding parameters, so it must blindly recover the encoder before decoding. In this paper we focus on the blind recognition of coding parameters of an encoder which uses convolutional codes as error-correction coding and propose a method to take advantage of the soft information in soft-decision situations.

Some previous literatures discussed the problem of blind recognition of convolutional codes. The authors of [1–3] developed recognition methods in noiseless context, including the rate 1/*n* and *k*/*n* codes, where *n* denotes the codeword length and *k* the dimension. These methods are not suitable for noisy environment. For the case of noisy context, some algorithms were proposed in recent years [4–7]. The algorithm proposed in [7], which we call a Gauss-Jordan elimination through pivoting (GJETP) based algorithm in this paper, completely solved the blind parameter recognition of *k*/*n* convolutional codes with low complexity and excellent recognition performances.

However, the previous works are all discussed in hard-decision situations. In modern communication systems, more and more soft-decision based algorithms are applied to improve the signal processing performances. For example, the soft-decision based decoding methods always have better performances than hard-decision situations [8–12]. Similarly, some soft-decision based blind recognition of block code parameters also outperforms the hard-decision one [13, 14].

In Section 2 of this paper, based on [6, 7] we propose a method to utilize the soft information to improve the recognition performances in soft-decision systems. And we give another two improvements about the recognition algorithm in Sections 3 and 4: (1) because the authors of [6, 7] did not give a normative algorithm to automatically identify the parameter *n* for a computer program, we propose a formalized scheme to optimize the recognition of *n*; (2) the authors of [6, 7] did not consider the synchronization of codewords while the synchronization positions are usually unknown in a noncooperative communication context; we propose to modify the algorithm to identify the codeword synchronization.

Besides, in Section 5 we propose a correlation attack method to meet very low signal-to-noise ratio (SNR) situations, which also includes both hard- and soft-decision algorithms. This method might require more computational time, but it can push the SNR limits of the GJETP-based algorithm proposed in [7]. And we propose some strategies to reduce the searching space according to the structural analysis of the dual codes.

Finally we show the efficiency of the proposed algorithm by computer simulations in Section 6 and conclude the paper in Section 7.

## 2. Utilizing the Soft Information

The details of the GJETP-based recognition of rate *k*/*n* convolutional encoder parameters are introduced in [7]. The algorithm includes three major sections:

1. identification of code length *n*;
2. identification of a dual code basis;
3. identification of the generator matrix.

In the first procedure, the authors of [7] propose to recognize parameter *n* using the following steps.

**Step 1**

Set the minimum and maximum value of *l*, that is, *l*_min⁡_ and *l*_max⁡_. Initialize *l* to be *l*_min⁡_.

**Step 2**

**Figure 1:**
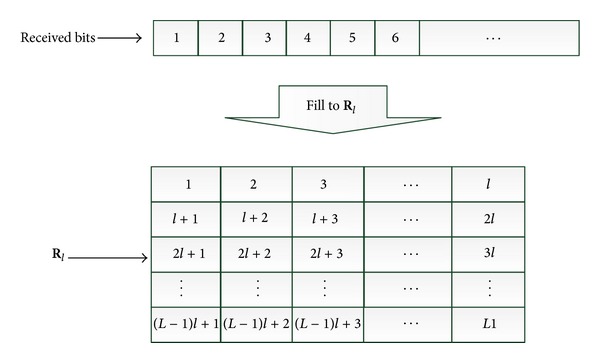
Generation of the observed matrix **R**_*l*_.

According to *l* and a sequence of received symbols, we generate an observed data matrix **R**_*l*_ as shown in Figure 1, the numbers in which denote the arriving order of the bits in the received data stream.

**Step 3**

Transform the matrix **R**_*l*_ to a lower triangular matrix **G**_*l*_ by GJETP (see [15] for details):

(1) $$\begin{array}{c} G_{l}=A_{l}R_{l}B_{l}. \end{array}$$

**Step 4**

Obtain the set **Z**_*l*_ as follows:

(2) $$\begin{array}{c} Z_{l}=\text{Card}\left\{i\in \left\{1,\ldots ,l\right\}\;|\;N_{l}\left(i\right)\leq \frac{L-l}{2}\gamma_{\text{opt}}\right\}, \end{array}$$

where *N*_*l*_(*i*) is the number of “1” in the lower part of the *i*th column in the matrix **G**_*l*_,  *γ*_opt_ is an optimal threshold [6, 7], and Card{*x*} denotes the cardinal of *x*. An *N*_*l*_(*i*) smaller than (*L* − *l*)*γ*_opt_/2 indicates that the *i*th column of **R**_*l*_ has a high probability to be dependent on the other columns.

**Step 5**

If *l* = *l*_max⁡_, execute Step 6. If *l* < *l*_max⁡_, let *l* = *l* + 1 and go back to Step 2.

**Step 6**

Output the gap between two consecutive nonzero cardinals, **Z**_*l*_, as the estimated codeword size $\hat{n}$. The principle of this estimation is that when *l* = *mn*(*m* ∈ **Z** ^+^) and *l* is larger than a parameter *n*_*a*_ (see [7] for details), some columns of **C**_*l*_ can be determined by other columns, where **C**_*l*_ = **R**_*l*_ + **E**__*l*__ and **E**_*l*_ is the error pattern corresponding to **R**_*l*_.

According to the GJETP algorithm, the reliability of upper part of **R**_*l*_ has larger influence on the successful detection of dependent columns in **R**_*l*_. So if we can make the upper part of **R**_*l*_ have lower errors, the algorithm can be improved. In hard-decision situations, we cannot determine which rows in **R**_*l*_ have lower probabilities to have error decision bits. But in soft-decision situations, we can examine the reliabilities of the rows in **R**_*l*_ according to the soft-decisions bits. Therefore, here we propose the following processing procedure inserted into the steps (between Step 2 and Step 3) mentioned above.

1. Fill the observed matrix **R**_*l*_ with the soft-decision bits. For each row in **R**_*l*_ we find the decision bit that has the lowest reliability and record its reliability value as the reliability of the row.
2. Arrange the rows of **R**_*l*_ according to each row's lowest reliability value to make the first row of **R**_*l*_ have the highest reliability, the second row have the second highest reliability, and so on.
3. Obtain the hard decisions of the bits in the rearranged **R**_*l*_ and continue Step 3 mentioned above.

After this processing, the upper rows of the rearranged **R**_*l*_ have higher reliabilities and therefore have lower probabilities to include error decision bits. So the probability of successful detection of dependent columns can be improved. And as the value of *L* rises, the improvement of the reliabilities of the upper part of **R**_*l*_ becomes more effective, so the recognition performance can be improved more obviously than the previous algorithm in [7]. After the estimation of *n*, we use Algorithms  1 and 2 in [7] to identify other coding parameters. Note that the parity check vector recognition procedure uses the GJETP algorithm too, so the proposed rearranged steps are also applied before each GJETP processing when recognizing the parity check vectors.

## 3. Formalizing the Estimation Algorithm of *n*

Note that, in Step 6 shown in the previous section, the authors of [7] only give the idea of the estimation of *n*. When the noise level is high, there exists a problem that the number of error decision bits is high and some cardinals **Z**_*l*_ corresponding to the values of *l* that equals *mn* are blank. So not all the gaps between two consecutive nonzero cardinals, **Z**_*l*_, equal *n*. In this case, we need a formalized algorithm to estimate the value of *n* more exactly. This can be done by simply searching all the gaps between two consecutive nonzero cardinals and find out which gap value appears mostly. The detailed algorithm steps are listed below.

(1) Let  **V**_*l*_ = [*l*_1_, *l*_2_,…, *l*_*s*_] be the vector that consists of the values of *l* corresponding to the detected nonzero cardinals **Z**_*l*_ in Step 6.
(2) Calculate the vector Δ**V**_*l*_ = [Δ*l*_1_, Δ*l*_2_,…, Δ*l*_*s*−1_], the elements of which are calculated by
  (3) $$\begin{array}{c} \Delta l_{i}\left(1\leq i\leq s-1\right)=l_{i+1}-l_{i}. \end{array}$$
(3) Initialize a vector **Q** with length *l*_max⁡_ to be overall zeros and for each value of *i* from 1 to *s* − 1, we let
  (4) $$\begin{array}{c} Q\left(\Delta l_{i}\right)=Q\left(\Delta l_{i}\right)+1. \end{array}$$
(4) Finally we find the maximum element in **Q** and output the corresponding gap value  Δ*l*_*i*_ to be the estimated codeword size $\hat{n}$.

After taking the previously mentioned searching method we can find the most probable gap between two consecutive nonzero cardinals, that is, the estimation of the code length. An example as follows further describes the searching procedure.

**Figure 2:**
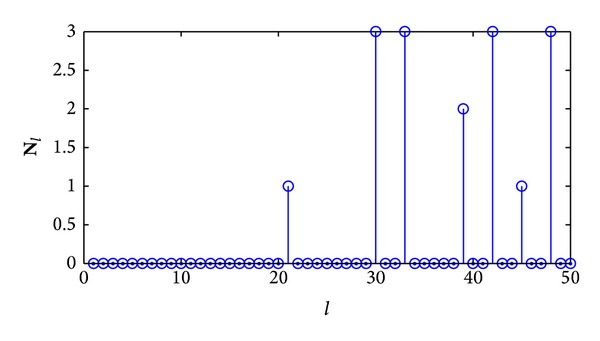
The recorded vector **N**_*l*_ based on low SNR.

Figure 2 shows the recorded vector **N**_*l*_ when recognizing a *C*(3, 2, 4) convolutional code based on correct synchronization positions with a low SNR (*E*_*s*_/*N*_0_ = 4 dB). From the figure we can see that not all the gaps between two consecutive nonzero elements in **N**_*l*_ equal the code length, 3. If we estimate the code length by the gap between the first and the second nonzero elements in **N**_*l*_, the estimation is not correct. Implementing the searching steps mentioned above, we can firstly obtain the vector **V**_*l*_ as follows:

(5) $$\begin{array}{c} V_{l}=\left[\begin{array}{ccccccc} 21, & 30, & 33, & 39, & 42, & 45, & 48 \end{array}\right]. \end{array}$$

Then we can calculate the vector Δ**V**_*l*_ according to **V**_*l*_ as follows:

(6) $$\begin{array}{c} \Delta V_{l}=\left[\begin{array}{cccccc} 9 & 3 & 6 & 3 & 3 & 3 \end{array}\right]. \end{array}$$

Furthermore, we can calculate the vector **Q** by (4). In the vector **Q**, we have **Q**(3) = 4, **Q**(6) = **Q**(9) = 1, and other elements in **Q** equal to zero. **Q**(3) is the maximum element in the vector **Q**, so, according to Step 4 mentioned above, we can estimate the code length:$\;\hat{n}=3$.

This example shows an implementation of the formalized algorithm proposed in this section to estimate the code length more exactly in a low SNR situation.

## 4. Recognition of Synchronization Positions

The GJETP-based dual code method proposed in [7] has good performances on convolutional encoder identifications. But unfortunately, the authors did not consider the codeword synchronization problem. In noncooperative context, the synchronization cannot usually be reached before the coding parameters are correctly estimated. The algorithm is discussed based on correct synchronization. In the case that the codeword synchronization position is unknown, we can but randomly choose a position in the received data stream to be the beginning of a codeword. The randomly chosen synchronization positions have low influence on the recognition of code length, but the recognition of key parameter *n*_*a*_ (the minimal length of the rows of matrix **R**_*l*_ so that **R**_*l*_ includes dependent columns; see [7] for details) and parity check vectors are not correct.

**Figure 3:**
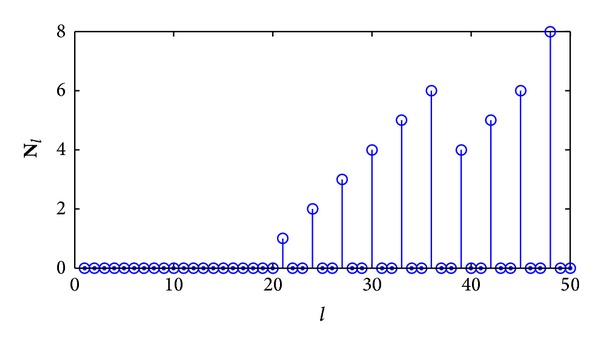
The recorded vector **N**_*l*_ based on correct synchronization.

**Figure 4:**
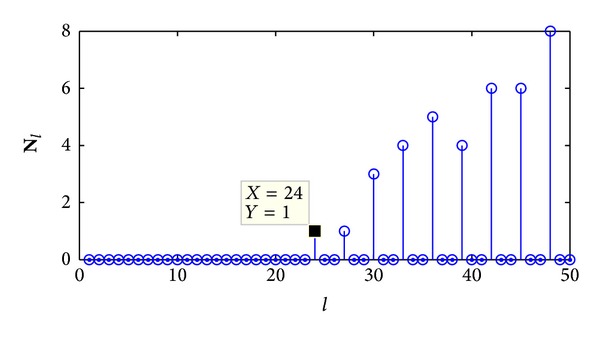
The recorded vector **N**_*l*_ based on incorrect synchronization.

Figure 3 shows the recorded vector **N**_*l*_ when recognizing a *C*(3, 2, 4) convolutional code based on correct synchronization positions. From the figure we can estimate the key parameter *n*_*a*_ = 21, which is accordant to the *C*(3, 2, 4) convolutional code. But if we choose an incorrect synchronization position, as shown in Figure 4, the first nonzero element in vector **N**_*l*_ appears at the position *l* = 24, so the key parameter *n*_*a*_ is estimated to be 24, which is a fault estimation. In order to obtain correct parity check vectors, we must firstly locate the correct synchronization positions.

We assume that when recognizing the code length we fill the matrix **R**_*l*_ from the *t*_0_th position from the received bit stream and the estimated code length and key parameter *n*_*a*_ are $\hat{n}$ and ${\hat{n}}_{a}$, respectively, and then we propose the following steps  to find the correct codeword synchronization.

**Step 1**

Let *τ* = *t*_0_ + 1, ${\overset{-}{n}}_{a}=n_{a}$, and $\overset{-}{\tau }=t_{0}$.

**Step 2**

Let $l={\hat{n}}_{a}-n$.

**Step 3**

Fill the matrix **R**_*l*_ with row length *l* from the *τ*th bit in the received data stream.

**Step 4**

Do the GJETP processing for **R**_*l*_ and calculate  **N**_*l*_, the number of dependent columns.

**Step 5**

If **N**_*l*_ > 0 and $l<{\overset{-}{n}}_{a}$, let ${\overset{-}{n}}_{a}=l$, $\overset{-}{\tau }=\tau$, and *l* = *l* − *n*, and go back to Step 3; otherwise, execute Step 6.

**Step 6**

If *τ* < *t*_0_ + *n* − 1, let *τ* = *τ* + 1 and go back to Step 2.

**Step 7**

Let ${\overset{-}{n}}_{a}$ and $\overset{-}{\tau }$ be the newly estimations of *n*_*a*_ and synchronization position.

The previous steps  corrected the codeword synchronization. For the following procedure of parity check matrix recognition, we use *τ* to be the synchronization position to fill the matrix **R**_*l*_ and replace *n*_*a*_ by ${\overset{-}{n}}_{a}$ in the algorithms.

## 5. Correlation Attack Method

### 5.1. Hard-Decision Situations

If the polynomial-based generator matrices of a *k*/*n* convolutional code and the dual code are **G**(*D*) and **H**(*D*), respectively, we have [16]

(7) $$\begin{array}{c} G\left(D\right)\times H\left(D\right)=0. \end{array}$$

According to the analysis of [7], **H**(*D*) has the following style:

(8) $$\begin{array}{c} H\left(D\right)=\left[\begin{array}{cccccc} h_{1,1}\left(D\right) & \cdots & h_{1,k}\left(D\right) & h_{0}\left(D\right) & & \\ \vdots & \ddots & \vdots & & \ddots & \\ h_{n-k,1}\left(D\right) & \cdots & h_{n-k,k}\left(D\right) & & & h_{0}\left(D\right) \end{array}\right], \end{array}$$

where *h*_*j*,*s*_(*D*)  (1 ≤ *j* ≤ *n* − *k*, 1 ≤ *s* ≤ *k*) is a polynomial, coefficients of which are all in GF(2):

(9) $$\begin{array}{c} h_{j,s}\left(D\right)=h_{j,s}\left(0\right)+h_{j,s}\left(1\right)D+\cdots +h_{j,s}\left(\mu^{⊥}\right)D^{\mu^{⊥}}. \end{array}$$

Parameter *μ* ^⊥^ in (7) is the memory of the dual code [6, 7].

*h*_0_(*D*) is also a polynomial on GF(2) as follows:

(10) $$\begin{array}{c} h_{0}\left(D\right)=h_{0}\left(0\right)+h_{0}\left(1\right)D+\cdots +h_{0}\left(\mu^{⊥}\right)D^{\mu^{⊥}}. \end{array}$$

And according to [7], for a realizable convolutional encoder, we have

(11) $$\begin{array}{c} h_{0}\left(0\right)=1. \end{array}$$

Based on (8)–(10), we can define the binary form of **H**(*D*) as follows:

(12) $$\begin{array}{c} H=\left[\begin{array}{ccccccc} H_{\mu^{⊥}} & H_{\mu^{⊥}-1} & \cdots & H_{0} & & & \\ & H_{\mu^{⊥}} & H_{\mu^{⊥}-1} & \cdots & H_{0} & & \\ & & H_{\mu^{⊥}} & H_{\mu^{⊥}-1} & \cdots & H_{0} & \\ & & & \ddots & \ddots & \ddots & \ddots \end{array}\right], \end{array}$$

where

(13) $$\begin{array}{c} H_{i}=\left[\begin{array}{cccccc} h_{1,1}\left(i\right) & \cdots & h_{1,k}\left(i\right) & h_{0}\left(i\right) & & \\ \vdots & \ddots & \vdots & & \ddots & \\ h_{n-k,1}\left(i\right) & \cdots & h_{n-k,k}\left(i\right) & & & h_{0}\left(i\right) \end{array}\right]. \end{array}$$

It is shown in (12) that each row of the matrix **H** is a shift of the following (*n* − *k*) × *n*(*μ* ^⊥^ + 1) matrix **H**_0_, which we call the basic check matrix of convolutional codes in this paper:

(14) $$\begin{array}{c} H_{0}=\left[\begin{array}{cccc} H_{\mu^{⊥}} & H_{\mu^{⊥}-1} & \cdots & H_{0} \end{array}\right]. \end{array}$$

So the recognition problem of a convolutional encoder is equivalent to the recognition of **H**_0_. We call the vectors in **H**_0_ the basic parity check vectors. According to the dual code principles, we can consider **H**_0_ to be a parity check matrix for (*μ* ^⊥^ + 1) consecutive codewords. That is to say, if a row vector **c** includes (*μ* ^⊥^ + 1) consecutive codewords, we have

(15) $$\begin{array}{c} c\times H_{0}^{T}=0. \end{array}$$

Furthermore, for a matrix **C** consisting of a number of such vectors, we have

(16) $$\begin{array}{c} C\times H_{0}^{T}=0. \end{array}$$

In this paper we say two vectors **c** and **h** are correlated if they have the same length *l* and

(17) $$\begin{array}{c} \overset{l}{\underset{i=1}{\sum }}c_{i}h_{i}=0, \end{array}$$

where the sum operator is defined in GF(2) and

(18) c=[c1c2⋯cl],h=[h1h2⋯hl].

One solution to the recognition of **H**_0_ is enumerating all possible length values *l* and listing all vectors with length *l* to see which vectors have likelihood to be the basic parity check vectors. This can be implemented by enumerating all possible length values *l*, creating the observed data matrix **R**_*l*_ with row length *l* (as shown in Figure 1), and trying to find the vectors which are correlated with most rows of **R**_*l*_. If we can find such vectors, the corresponding *l* can be considered to be a possible estimation of *ω*, which is defined to be the row length of the basic check matrix **H**_0_:

(19) $$\begin{array}{c} \omega =n\left(\mu^{⊥}+1\right). \end{array}$$

Furthermore, we can estimate the code length *n* and dual code memory length *μ* ^⊥^ by investigating the factors of the estimated *ω*.

This scheme takes a very long elapsed time. Here we propose some principles to reduce the searching space.

1. Reduce the candidates of *l*.

According to (19), the parameter  *ω*  is a product of two integers which are all larger than 1. Therefore, we can drop the prime values while searching. So we just need to enumerate the values of *l* from composite numbers.

1. Reduce the candidates of basic parity check vectors.

We can set the first and the last bit of each candidate vector to be 1. Hence, we just need to enumerate the combinations of the middle (*l* − 2) bits for each* l*-length vector.

In the noisy environment, not all the rows in **R**_*l*_ are correlated with the basic parity check vectors. **R**_*l*_ can be written as follows:

(20) $$\begin{array}{c} R_{l}=C_{l}+E_{l}, \end{array}$$

where **E**_*l*_ is the error pattern corresponding to the elements in **R**_*l*_ and the elements in **C**_*l*_ are the original encoded (noiseless) bits of the received bits in **R**_*l*_. If *l* = *n*(*μ* ^⊥^ + 1) and no error exists in the received stream, that is, **E**_*l*_ = 0, according to (16), we have

(21) $$\begin{array}{c} R_{l}\times H_{0}^{T}=0. \end{array}$$

In most noisy environments, because of the existence of error bits, (21) is not always true even if *l* equals *n*(*μ* ^⊥^ + 1). So we cannot determine whether a vector **h** is correlated with row vectors of **R**_*l*_ by checking the equation **R**_*l*_ × **h** ^*T*^ = 0. Instead, we need to compute the likelihood of a vector **h** to be correlated with **R**_*l*_ and an appropriate threshold to determine whether the vector **h** can be considered to be a basic check vector. We now deduce the threshold below.

For a given data matrix **R**_*l*_ and vector **h**, we denote by **q** a vector calculated by **R**_*l*_ × **h** ^*T*^ as follows:

(22) $$\begin{array}{c} q=R_{l}\times h^{T}. \end{array}$$

Equation (22) is defined in GF(2), so the elements in vector **q** are from the set {0, 1}. And we denote by *wt*(**q**) the Hamming weight of the vector **q**. In noisy environment, the expectation of *wt*(**q**) can be calculated as follows:

(23) $$\begin{array}{c} E\left[wt\left(q\right)\right]=L\left[1-\text{Pr}\left(c\times h^{T}=0\right)\right], \end{array}$$

where *L* is the number of rows of the observed data matrix **R**_*l*_ and **c** is any possible form of the rows of **R**_*l*_. Pr(**c** × **h** ^*T*^ = 0) is the probability of **c** × **h** ^*T*^ = 0. If the matrix **R**_*l*_ is filled with correct parameters, that is, *l* = *n*(*μ* ^⊥^ + 1)  , the first bit filled into **R**_*l*_ is the beginning of a codeword, and the vector **h** is a valid check vector, Pr(**c** × **h** ^*T*^ = 0) equals the probability that the vector **c** has even error bits. So we can calculate the probability Pr(**c** × **h** ^*T*^ = 0) as follows:

(24) $$\begin{array}{c} \text{Pr}\left(c\times h^{T}=0\right)=\overset{\left\lfloor w/2\right\rfloor }{\underset{i=0}{\sum }}\left(\begin{array}{c} w\\ 2i \end{array}\right)\tau^{2i}{\left(1-\tau \right)}^{w-2i}=\frac{1+{\left(1-2\tau \right)}^{w}}{2}, \end{array}$$

where *w* is the Hamming weight of **h** and  *τ* is the channel transition probability. According to (23) and (24), we have

(25) $$\begin{array}{c} E_{1}=E\left(wt\left(q\right)\right)=\frac{1-{\left(1-2\tau \right)}^{w}}{2}L. \end{array}$$

According to binomial-distribution theories, we can calculate the variance of *wt*(**q**) as follows:

(26) $$\begin{array}{c} D_{1}=D\left(wt\left(q\right)\right)=\frac{1-{\left(1-2\tau \right)}^{2w}}{4}L. \end{array}$$

If the data matrix **R**_*l*_ is based on incorrect parameters or **h** is not a valid check vector, the probability Pr(**c** × **h** ^*T*^ = 0) is

(27) $$\begin{array}{c} \text{Pr}\left(c\times h^{T}=0\right)=\text{Pr}\left(c\times h^{T}=1\right)=\frac{1}{2}. \end{array}$$

Therefore, the expectation and variance of *wt*(**q**) are

(28) $$\begin{array}{c} E_{2}=E\left(wt\left(q\right)\right)=\frac{1}{2}L,\\ D_{2}=D\left(wt\left(q\right)\right)=\frac{1}{4}L. \end{array}$$

So we propose the threshold *δ* based on *λ*-standard deviation principle as follows:

(29) δ=(E1+λD1)+(E2−λD2)2=([((1−(1−2τ)w)/2)L+λ((1−(1−2τ)2w)/4)L]  +((1/2)−λ(1/4)L))(2)−1=L2[1−(1−2τ)w2]+λL4[1−(1−2τ)2w−1].

Experimentally, we suggest choosing the parameter *λ* between 6 and 8 to get a good recognition result.

In (29), we must ensure that

(30) $$\begin{array}{c} \left(E_{1}+\lambda \sqrt{D_{1}}\right)<\left(E_{1}-\lambda \sqrt{D_{2}}\right). \end{array}$$

So the number of rows of the observed data matrix **R**_*l*_ should meet the following condition:

(31) $$\begin{array}{c} L>{\left[\frac{\lambda \sqrt{1-{\left(1-2\tau \right)}^{2w}}+1}{{\left(1-2\tau \right)}^{w}}\right]}^{2}. \end{array}$$

The threshold *δ* and corresponding condition of *L* are based on the prior information about the noise level, that is, the channel transition probability *τ*. If such prior information is unknown, the calculation of (29) and (31) cannot be done. In this situation, we propose to set the threshold *δ* to be *L*/10 and let

(32) $$\begin{array}{c} \left(\frac{1}{2}L-\lambda \sqrt{\frac{1}{4}L}\right)>\delta =\frac{L}{10}. \end{array}$$

Therefore, *L* should meet the following condition:

(33) $$\begin{array}{c} L>{\left(\frac{5}{4}\lambda \right)}^{2}. \end{array}$$

The threshold *δ* = *L*/10 is just an experimental value. If no parity check vector can be found based on such a threshold, we can increase the value of *δ* and redo the searching procedure. If *δ* = *μ*/*θ*  (*θ* > 2), we must choose *L* such that

(34) $$\begin{array}{c} \left(\frac{1}{2}L-\lambda \sqrt{\frac{1}{4}L}\right)>\delta =\frac{L}{\theta }; \end{array}$$

that is,

(35) $$\begin{array}{c} L>{\left(\frac{\theta }{\theta -2}\lambda \right)}^{2}. \end{array}$$

To implement the algorithm automatically by a computer program, we propose the following procedure to recognize the parameter  *ω*  defined by (19) and the codeword synchronization position *t*_0_ as follows.

**Step 1**

Set the searching range of *ω*; that is, set *ω*_min⁡_ and *ω*_max⁡_.

**Step 2**

List all the composite numbers between *ω*_min⁡_ and *ω*_max⁡_ to form a set

(36) $$\begin{array}{c} \left\{\begin{array}{cccc} \omega_{1} & \omega_{2} & \cdots & \omega_{\varepsilon } \end{array}\right\}, \end{array}$$

where *ω*_1_ < *ω*_2_ < ⋯<*ω*_*ε*_,  *ε*  is the number of composite numbers between *ω*_min⁡_ and *ω*_max⁡_.

**Step 3**

Let  *i* = 1.

**Step 4**

Let  *l* = *ω*_*i*_.

**Step 5**

Let *t* = 1; fill the data matrix **R**_*l*_ with the received data. The first bit filled into **R**_*l*_ is the *t*th bit in the received stream.

**Step 6**

Let *j* = 1.

**Step 7**

Create a vector:

(37) $$\begin{array}{c} h=\left[\begin{array}{ccccc} h_{1} & h_{2} & \cdots & h_{l-1} & h_{l} \end{array}\right], \end{array}$$

where *h*_1_ = *h*_*l*_ = 1 and *h*_2_ *h*_3_ ⋯ *h*_*l*−1_ is the binary form of *j* with length *l* − 2.

**Step 8**

Calculate the vector **q** by (22) and denote by *wt*(**q**) the Hamming weight of **q**. If *wt*(**q**) < *δ*, stop the searching and output *t* and *l* to be the estimation of *t*_0_ and *ω*; that is, let ${\hat{t}}_{0}=t$ and $\hat{\omega }=l$. Besides, record **h** as **h**_*x*_.

**Step 9**

If *j* < 2^*l*−2^ − 1, let *j* = *j* + 1 and go back to Step 7. Otherwise, execute Step 10.

**Step 10**

If *t* < *n*_max⁡_, let *t* = *t* + 1 and go back to Step 6. Otherwise, execute Step 9. *n*_max⁡_ is the maximum of possible codeword length. *n*_max⁡_ can be set to the maximum factor of* l* (except *l* itself).

**Step 11**

If *i* < *ε*, go back to Step 4. Otherwise, execute Step 12.

**Step 12**

End the searching.

If we can stop the searching from Step 8, we can recognize the codeword length *n* based on ${\hat{t}}_{0}$, $\hat{\omega }$, and **h**_*x*_. According to (19), the code length *n* is a factor of *ω*. So we propose to estimate *n* by the following steps.

**Step 1**

List all the factors of *ω* (expect 1 and *ω* itself) to form a set

(38) $$\begin{array}{c} \left\{\begin{array}{cccc} n_{1} & n_{2} & \cdots & n_{\sigma } \end{array}\right\}, \end{array}$$

where *n*_1_ < *n*_2_ < ⋯<*n*_*σ*_, *σ* is the number of the factors of *ω* (expect 1 and *ω* itself); let  *l* = *ω*.

**Step 2**

Let *i* = 1.

**Step 3**

Let *n* = *n*_*i*_.

**Step 4**

Fill the data matrix **R**_*l*_ as follows:

(39) $$\begin{array}{c} R_{l}=\left(\begin{array}{c} c_{1}\\ c_{2}\\ \vdots \\ c_{L} \end{array}\right), \end{array}$$

where

(40) c1=[c1⋯cn ∣ cn+1⋯c2n ∣ ⋯ ∣ cμ⊥n+1⋯c(μ⊥+1)n],c2=[cn+1⋯c2n ∣ c2n+1⋯c3n ∣ ⋯ ∣ c(μ⊥+1)n+1⋯c(μ⊥+2)n],c3=[c2n+1⋯c3n ∣ c3n+1⋯c4n ∣ ⋯ ∣ c(μ⊥+2)n+1⋯c(μ⊥+3)n],⋮⋮

where *c*_*i*_  (*i* ≥ 1) is the $\left({\hat{t}}_{0}+i-1\right)$th bit in the received data stream and $\mu^{⊥}=\hat{\omega }/n-1$.

**Step 5**

Calculate the vector **q** by **q** = **R**_*l*_ × **h**_*x*_^*T*^ and get *wt*(**q**), the Hamming weight of **q**.

**Step 6**

If *wt*(**q**) < *δ*, stop the searching and output the current *n* to be the estimated codeword length:$\;\hat{n}=n$. Otherwise, execute Step 7.

**Step 7**

If *i* < *σ*, let *i* = *i* + 1 and go back to Step 3. Otherwise, execute Step 8.

**Step 8**

End the searching.

After the estimation of *n*, we can simply get the estimation of *μ* ^⊥^ by

(41) $$\begin{array}{c} {\hat{\mu }}^{⊥}=\frac{\hat{\omega }}{\hat{n}}-1. \end{array}$$

Finally, we search all the basic check vectors to recover the basic check matrix **H**_0_. According to (13) and (14), we can estimate the encoder parameter *k* and the  *n* − *k*  parity check vectors by the following steps.

**Step 1**

Let *k* = 1.

**Step 2**

Let *s* = 1.

**Step 3**

Create a vector **x**_*s*_ as follows:

(42) $$\begin{array}{c} x_{s}=\left[\begin{array}{cccccccccc} c_{1} & \cdots & c_{k} & c_{k+s} & c_{\hat{n}+1} & \cdots & c_{\hat{n}+k} & c_{\hat{n}+k+s} & c_{2\hat{n}+1} & \cdots \end{array}\right], \end{array}$$

where the definition of  *c*_*i*_  (*i* ≥ 1)  is the same as (40).

**Step 4**

Let *l* = (*k* + 1)(*μ* ^⊥^ + 1) and fill the elements in **x**_*s*_ into the matrix **R**_*l*_ with row length *l*.

**Step 5**

Enumerate all vectors **h** with length *l*, where the last element of **h** is 1, and calculate *wt*(**q**), where **q** = **R**_*l*_ × **h**_*x*_^*T*^. If any vector **h** can make *wt*(**q**) < *δ*, record ${\hat{h}}_{s}=h$ and execute Step 6. Otherwise, execute Step 8.

**Step 6**

If $s<\hat{n}-k$, let  *s* = *s* + 1 and go back to Step 3. Otherwise, execute Step 7.

**Step 7**

Stop the searching, and output $\hat{k}=k$ and the recorded $\hat{n}-\hat{k}$ vectors ${\hat{h}}_{1},{\hat{h}}_{2},\ldots ,{\hat{h}}_{\hat{n}-\hat{k}}$.

**Step 8**

If $k<\hat{n}-1$, let  *k* = *k* + 1 and go back to Step 2. Otherwise, execute Step 9.

**Step 9**

End the searching.

If such recognition procedure can successfully output$\;\hat{n}-\hat{k}\;$vectors$\;{\hat{h}}_{1},{\hat{h}}_{2},\ldots ,{\hat{h}}_{\hat{n}-\hat{k}}$, we can finally recover the basic parity check matrix **H**_0_. We write the vectors ${\hat{h}}_{1},{\hat{h}}_{2},\ldots ,{\hat{h}}_{\hat{n}-\hat{k}}$ as follows:

(43) h^1=[h1,1⋯h1,k^h1,k^+1 ∣ h1,(k^+1)+1⋯h1,(k^+1)+k^h1,2(k^+1) ∣ ⋯ ∣ h1,μ^⊥(k^+1)+1⋯h1,μ^⊥(k^+1)+k^h1,(μ^⊥+1)(k^+1)],h^2=[h2,1⋯h2,k^h2,k^+1 ∣ h2,(k^+1)+1⋯h2,(k^+1)+kh2,2(k^+1) ∣ ⋯ ∣ h2,μ⊥(k^+1)+1⋯h2,μ^⊥(k^+1)+k^h2,(μ^⊥+1)(k^+1)],⋮h^n^−k^=[hn^−k^,1⋯h1,k^h1,k^+1 ∣ hn^−k^,(k^+1)+1⋯hn^−k^,(k^+1)+k^hn^−k^,2(k^+1) ∣ ⋯ ∣ hn^−k^,μ^⊥(k^+1)+1⋯hn^−k^,μ^⊥(k^+1)+k^hn^−k^,(μ^⊥+1)(k^+1)],

and then the parity check matrix **H**_0_ can be estimated as follows:

(44) H^0=[h^1,1⋯h^1,kh^1,k+1h^1,k+2⋯h^1,2k+1h^1,2k+2h^1,k+2⋮⋱⋮⋱⋮⋱⋮⋱⋮h^n−k,1⋯h^n−k,kh^n−k,k+1h^n−k,k+2⋯h^n−k,2k+1h^n−k,2k+2h^n−k,k+2⋯⋯…].

### 5.2. Soft-Decision Situations

In hard-decision situations, we calculate *wt*(**q**) by evaluating how many vectors in **R**_*l*_ are correlated with a candidate parity check vector **h**. In soft decisions, we can utilize the soft output from the receiver to evaluate the reliability of each decision bit. Therefore, we can calculate the probability of each row in **R**_*l*_ to be correlated with **h**. For example, if a communication system uses BPSK demodulation in an additive-white-Gaussian-noise (AWGN) channel, according to the analysis in [14, 15], we can calculate the probability of **c** × **h** ^*T*^ = 1 and **c** × **h** ^*T*^ = 0 for given vectors **c** and **h** as follows:

(45) Pr(c×hT=0)=12+12∏j=1wtanh(cujσ2)Pr(c×hT=1)=12−12∏j=1wtanh⁡(cujσ2),

where *σ* is the noise variance, *w* is the Hamming weight of **h**, *u*_*j*_ is the *j*th nonzero position in **h**, and *c*_*u*_*j*__ is the  *u*_*j*_th element in vector** c**. Based on this, *wt*(**q**) is calculated as follows:

(46) $$\begin{array}{c} wt\left(q\right)=\overset{L}{\underset{i=1}{\sum }}P_{r}\left(c_{i}\times h^{T}=1\right), \end{array}$$

where **c**_*i*_ denotes the  *i*th row in **R**_*l*_.

## 6. Simulation Results

In this section we show the simulation results of the blind recognition of the convolutional coding parameters by utilizing the method introduced in this paper. The simulations include three parts corresponding to our proposed recognition algorithm on different noise level and different observed matrix size *L*. In the simulations we assume that the signal is transmitted on a binary symmetry channel (BSC) which is corrupted by AWGN.

**Figure 5:**
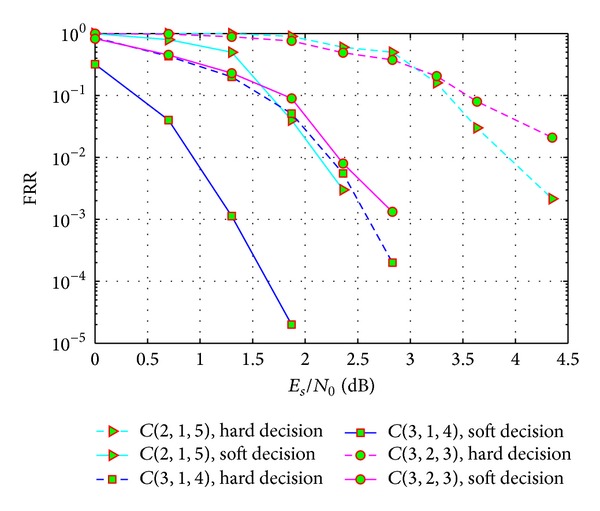
Recognition performances of GJETP method.

For the GJETP-based recognition method, we show the false recognition ratios (FRR) in Figure 5 for several convolutional codes in different channel conditions when the observed window size *L* = 200. We compare the proposed soft-decision based method with the hard-decision based algorithm proposed in [7]. We can see from the simulation results that when the soft information is introduced into the recognition algorithm, the recognition performances can be improved obviously. And as the SNR rises, the FRR curves descend more rapidly on soft-decision algorithm.

**Figure 6:**
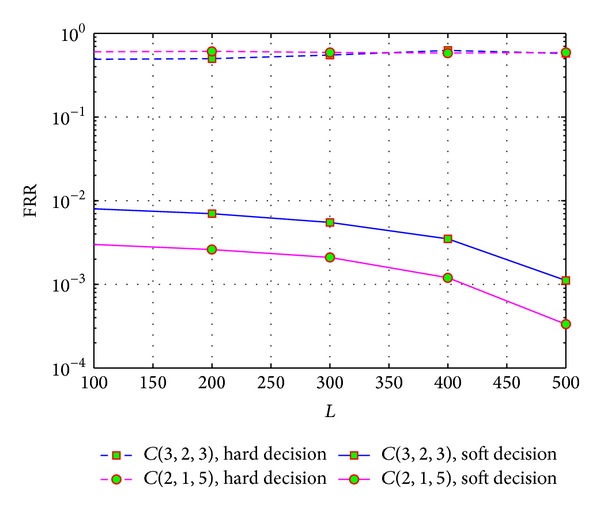
Recognition performances on different size of **R**_*l*_ for soft and hard decisions.

Figure 6 shows the recognition performance of two convolutional codes for different observed matrix size, when the SNR *E*_*s*_/*N*_0_ = 2.36 dB. It shows that the soft information can help to make the FRR descend more rapidly when *L* is rising. That is to say, in soft-decision situations, we can improve the recognition performance by increasing the number of rows in the data matrix of **R**_*l*_, while the hard-decision based algorithm cannot.

**Figure 7:**
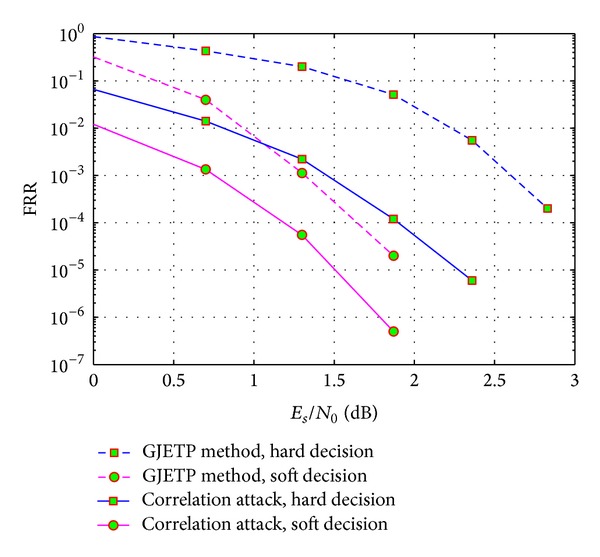
Comparison of GJETP method and correlation attack.

In Figure 7, we compare the performances of convolutional encoder recognition by GJETP method and correlation attack while recognizing the coding parameters of *C*(2, 1, 3). GJETP method is based on Gauss elimination on **R**_*l*_, so the influences of error bits are easily diffused during Gauss eliminating. Therefore, the fault-tolerance of GJETP method is limited. Correlation attack method can avoid this problem, so the recognition performance can be improved in low SNR situations.

## 7. Conclusions

This paper proposes the methods of utilizing soft information to improve the recognition performance of convolutional encoder parameters. And we propose a formalized estimation of the parameter *n* and synchronization positions. When introducing the soft information the recognition performance can be obviously improved and the simulations show the efficiency of the proposed methods. Besides, we propose a new algorithm to recover the basic parity check matrix by correlation attack. Although this method takes longer elapsed time, it can push the SNR limits of the GJETP method, and some principles are proposed to reduce the searching space. If the channel quality is well, the GJETP method has advantages on short computational delay. For a worse channel quality such that the GJETP does not work, correlation attack method has a significant advantage on its higher fault-tolerance.
